# Endogenous Endophthalmitis: A 10-Year Review of Cases on the East Coast of Malaysia

**DOI:** 10.7759/cureus.60132

**Published:** 2024-05-12

**Authors:** Amirul Hasbi, Mohd Nazri Mohd Nafeez, Evelyn Tai, Azhany Yaakub, Ismail Shatriah

**Affiliations:** 1 Department of Ophthalmology and Visual Sciences, School of Medical Sciences, Universiti Sains Malaysia, Kubang Kerian, MYS; 2 Department of Ophthalmology, Hospital Sultanah Nur Zahirah, Kuala Terengganu, MYS; 3 Department of Ophthalmology, Hospital Raja Perempuan Zainab II, Kota Bharu, MYS

**Keywords:** diabetes mellitus, liver abscess, klebsiella pneumoniae, south east asia, bacteria, intravitreal injection, vitrectomy, endogenous endophthalmitis

## Abstract

Purpose

Our study aimed to describe the clinical profile of endogenous endophthalmitis, focusing on patient demographics, infection sources, microbial profiles, clinical outcomes, and factors affecting the final visual outcome.

Methods

A retrospective review was performed on data from 68 eyes of 60 patients diagnosed with endogenous endophthalmitis and hospitalized in two tertiary hospitals on the East Coast of Peninsular Malaysia from January 2011 to December 2020. The analysis encompassed evaluating patient age, gender, laterality, risk factors, infection origins, presenting and final visual acuity, microbial results, treatment responses, and factors affecting final visual outcomes.

Results

The average age of the patient cohort was 54.9 ± 13.3 years. Females were more likely to have endogenous endophthalmitis than males (33, 55.0% vs. 27, 45.0%). Twenty-seven patients (45.0%) presented with endogenous endophthalmitis in the left eye, while 25 patients (41.7%) had it in the right eye, and eight patients (13.3%) had bilateral involvement. Most patients had underlying predisposing conditions, predominantly diabetes mellitus (53, 88.3%). Infection sources were identified in 42 patients (70.0%), out of which urinary tract infections account for the majority (11, 18.3%). *Klebsiella *species(14, 22.7%) were the leading pathogens and were significantly associated with liver abscess cases. In this series, the majority of patients had poor presenting and final visual acuity of worse than 3/60 (56, 82.4% and 53, 77.9%, respectively). Thirty-six eyes (52.9%) underwent vitrectomy, resulting in only four eyes (11.11%) achieving final visual acuity better than 6/12. Presenting visual acuity was identified as the factor contributing to the blind final visual outcome (*r* = 0.707, *p* < 0.001).

Conclusion

Females were found to be more commonly affected by endogenous endophthalmitis than males. *Klebsiella* species were the most commonly isolated microorganisms and were typically associated with liver abscesses. Urinary tract infection was the most common predisposing factor. A majority of the patients had poor presenting and final visual acuity, in which poor visual acuity is a significant indicator of blind visual outcomes.

## Introduction

Endogenous endophthalmitis is an aggressive eye infection that can lead to blindness. It occurs when infectious agents spread through the bloodstream from another part of the body, typically affecting individuals with weakened immune systems or prolonged use of medical devices. Endogenous endophthalmitis is uncommon, representing only two to eight percent of endophthalmitis cases [1-7]. Normally, the blood-ocular barrier (BOB) serves as a defense mechanism against invading pathogens. In endogenous endophthalmitis, inflammation compromises the BOB, allowing microorganisms to infiltrate the uveal tract or retinal circulation, leading to tissue damage. This inflammatory process often results in severe intraocular tissue destruction and carries a dismal prognosis for most patients.

Available data on endogenous endophthalmitis have been reported in Asia [1,6,8,9]. To date, limited data are available on the demographic characteristics of endogenous endophthalmitis in Southeast Asia, particularly involving multicenter analysis. This study aims to investigate the demographic and clinical characteristics, microbial profiles, treatment strategies, clinical outcomes of endogenous endophthalmitis, and factors associated with the final visual outcome in the tertiary eye centers on the East Coast of Malaysia.

## Materials and methods

A retrospective review was conducted on the medical records of patients diagnosed with endogenous endophthalmitis and treated between January 2011 and December 2020 at the Sultanah Nur Zahirah Hospital and Raja Perempuan Zainab II Hospital, in the states of Terengganu and Kelantan, respectively. Both are tertiary centers in the East Coast region of Malaysia. The study was conducted in accordance with the Declaration of Helsinki.

Endogenous endophthalmitis is defined as a severe intraocular infection that results from the hematogenous spread of microorganisms to the eye from a distant focus of infection [10]. The diagnostic criteria for endogenous endophthalmitis typically include a combination of presenting symptoms and clinical indicators. Patients with endogenous endophthalmitis may present with symptoms such as eye pain, redness, swelling, and reduced vision [11]. Clinical indicators that suggest endogenous endophthalmitis include lid edema, conjunctival chemosis, corneal haziness, hypopyon (pus in the anterior chamber), vitritis (inflammation of the vitreous), and positive culture sensitivity without evidence of an external cause [11]. All patients who fulfilled the diagnostic criteria for endogenous endophthalmitis were included in our study. Patients with a history of ocular trauma, recent ocular surgery within one year of onset, signs of primary external ocular infection, or referred cases from other tertiary eye centers were excluded from the study.

Demographic data and a comprehensive medical history, including both presenting complaints and underlying medical conditions, were obtained from the medical records. Patients’ ocular findings, including the presenting and final visual acuity, ocular signs, ultrasound B-scan examination, culture and sensitivity results, and treatment received, which include intravenous and intravitreal antibiotic injections and vitrectomies, were included in the data collection. Presenting and final visual acuity are defined as best corrected visual acuity (BCVA) at the initial presentation and at six months post-treatment, respectively. Improvement in visual acuity is defined as recovering more than one line on the Snellen chart compared to the baseline visual acuity. Deterioration of visual acuity is indicated by a worsening of more than one line on the Snellen chart compared to the baseline visual acuity. Static status occurs when there is no change in visual acuity from the baseline. We define blindness post-treatment when visual acuity is worse than 3/60 in accordance with the WHO definition of blindness.

Statistical analysis was performed using IBM SPSS Statistics for Mac, Version 28.0 (IBM Corp., Armonk, NY). Descriptive statistics were utilized for numerical variables (mean and standard deviation), and categorical data were expressed as frequency (n) and percentage. Simple and multiple logistic regressions were used to pinpoint factors correlated with blindness. The association between *Klebsiella* endophthalmitis and liver abscess was assessed using the Pearson chi-square test. Pearson's correlation was employed to determine the correlation between presenting visual acuity and final visual acuity.

## Results

Table 1 describes 68 eyes of 60 patients diagnosed with endogenous endophthalmitis, with unilateral involvement observed in 52 patients (86.7%) and bilateral involvement in eight patients (13.3%). Of the 60 patients, there were 27 males (45.0%) and 33 females (55.0%), with an average age of 54.9 ± 13.3 years at diagnosis (ranging from 21 to 83 years). Notably, unilateral involvement of the left eye (27, 45.0%) was more prevalent than that of the right eye (25, 41.7%). Among the patients, 58 (96.7%) had at least one predisposing factor to infection, most commonly diabetes mellitus, which affected 53 patients (88.3%).

**Table 1:** Clinical characteristics of patients (n = 60) DM, diabetes mellitus; ESRF, end-stage renal failure; F, female; HM, hand movement; HPT, hypertension; IHD, ischemic heart disease; LE, left eye; M, male; NPL, non-perceptive to light; PL, perceptive to light; RE, right eye; SABE, subacute bacterial endocarditis; UTI, urinary tract infection; VA, visual acuity

No.	Sex (M/F)	Age (Year)	Medical comorbidities	Isolates	Systemic infection	Laterality	Vitrectomy (Y/N)	Initial VA	Final VA
1	M	61	DM, HPT	No growth	Unknown	LE	Yes	HM	NPL
2	F	53	DM	Klebsiella pneumoniae	Parotid abscess	LE	Yes	HM	PL
3	F	60	HPT, ESRF	Klebsiella pneumoniae	Liver abscess	LE	Yes	1/60	6/24
4	M	41	DM	Klebsiella pneumoniae	Pneumonia	RE	Yes	PL	NPL
						LE	Yes	HM	NPL
5	F	21	HPT	No growth	Unknown	RE	Yes	HM	HM
6	M	50	DM	Staphylococcus aureus	Back carbuncle	RE	Yes	6/36	6/9
						LE	Yes	6/36	6/6
7	F	65	DM, HPT	Klebsiella pneumoniae	UTI	LE	Yes	HM	NPL
8	F	58	DM	No growth	Unknown	LE	Yes	PL	NPL
9	F	55	DM	Klebsiella pneumoniae	Liver abscess	RE	Yes	HM	6/9
						LE	Yes	HM	NPL
10	F	59	DM	No growth	Pneumonia	LE	Yes	PL	HM
11	F	30	DM	Klebsiella pneumoniae	Liver abscess	LE	Yes	6/9	4/60
12	F	65	DM, HPT	No growth	Unknown	LE	No	HM	HM
13	M	82	HPT	No growth	Thigh abscess	LE	No	NPL	NPL
14	F	83	HPT	Pseudomonas aeruginosa	Liver abscess	RE	No	NPL	NPL
15	M	40	DM, IHD	Candida albicans	Meningoencephalopathy	RE	No	1/60	6/36
						LE	No	1/60	6/36
16	M	62	DM	No growth	Unknown	LE	No	NPL	NPL
17	M	55	DM, HPT	No growth	SABE	LE	No	HM	NPL
18	M	66	DM	Klebsiella pneumoniae	Pneumonia	RE	No	PL	NPL
						LE	No	NPL	NPL
19	M	56	DM, HPT	Klebsiella pneumoniae	UTI	RE	No	CF	6/9
						LE	No	6/9	6/9
20	F	23	DM	No growth	Unknown	LE	No	6/9	6/12
21	F	71	DM, HPT	No growth	Unknown	LE	No	HM	NPL
22	M	72	DM	Aspergillus	Unknown	RE	No	PL	NPL
23	M	42	DM, HPT	No growth	Unknown	RE	No	NPL	NPL
24	F	44	DM, HPT	Staphylococcus aureus	Pneumonia	LE	No	HM	1/60
25	F	65	DM	Staphylococcus aureus	Gluteal abscess	RE	No	HM	HM
26	M	80	DM. ESRF	Pseudomonas aeruginosa	Pneumonia	RE	No	HM	NPL
27	M	58	DM	Staphylococcus aureus	Elbow abscess	RE	No	CF	3/60
28	M	52	DM, HPT	Staphylococcus aureus	Forearm abscess	RE	No	NPL	NPL
						LE	No	NPL	NPL
29	F	47	DM, HPT	Staphylococcus aureus	Leg abscess	LE	No	6/9	6/6
30	F	56	DM	Staphylococcus aureus	Knee septic arthritis	RE	No	HM	PL
						LE	No	PL	NPL
31	F	38	DM, HPT	No growth	Unknown	LE	No	NPL	NPL
32	M	57	HPT	No growth	UTI	LE	No	HM	HM
33	F	44	DM, HPT	No growth	Perinephric abscess	RE	No	6/9	6/9
34	M	68	DM	No growth	Unknown	RE	No	NPL	NPL
35	F	56	DM, HPT	Enterobacter cloacae	Unknown	RE	Yes	HM	HM
36	M	30	HPT	Staphylococcus aureus	Unknown	RE	Yes	6/60	HM
37	M	40	DM	Klebsiella pneumoniae	Liver abscess	RE	Yes	HM	NPL
38	F	69	DM, HPT	Escherichia coli	UTI	RE	No	6/60	NPL
39	M	71	DM	Staphylococcus aureus	Pneumonia	RE	Yes	HM	HM
40	M	47	DM	Klebsiella pneumoniae	Unknown	RE	Yes	CF	NPL
41	M	52	DM	No growth	UTI	LE	Yes	CF	6/24
42	F	62	DM	Acinetobacter lwoffii	Unknown	RE	Yes	HM	NPL
43	M	49	DM	Klebsiella pneumoniae	Pneumonia	LE	Yes	HM	NPL
44	F	47	DM	Klebsiella ozanae	Unknown	LE	Yes	PL	NPL
45	F	59	DM	Escherichia coli	UTI	LE	Yes	PL	NPL
46	F	63	DM	Pseudomonas aerugenosa	Pyonephrosis	LE	Yes	HM	HM
47	F	56	DM, HPT, ESRF	No growth	Pneumonia	RE	Yes	HM	NPL
48	F	53	NKMI	Klebsiella pneumoniae	Renal abscess	RE	Yes	6/36	NPL
49	M	67	DM	Candida albicans	UTI	RE	Yes	6/36	6/36
50	M	67	DM	Candida albicans	UTI	LE	Yes	CF	HM
51	M	41	DM	No growth	Liver abscess	RE	Yes	HM	HM
52	M	51	NKMI	No growth	Thigh abscess	LE	Yes	HM	2/60
53	F	59	DM	Escherichia coli	UTI	RE	Yes	6/60	NPL
54	M	51	DM	Staphylococcus aureus	Back abscess	RE	No	CF	1/60
55	F	32	DM	Klebsiella pneumoniae	Liver abscess	LE	Yes	HM	NPL
56	F	50	DM	Escherichia coli	Unknown	LE	Yes	PL	PL
57	F	54	DM	No growth	Unknown	RE	Yes	HM	NPL
58	F	65	DM, HPT	Staphylococcus aureus	UTI	LE	No	HM	NPL
59	F	62	DM, HPT, ESRF	Klebsiella pneumoniae	UTI	RE	No	HM	HM
60	F	63	HPT	No growth	Unknown	RE	Yes	HM	NPL

Table 2 shows that 42 patients (70%) had identifiable sources of infection, with genitourinary infection being the most frequent (14, 23.3%), out of which 11 cases (18.3%) were urinary tract infections. Other sources of infection included skin and lung infections in eight patients each (13.3%) and liver abscesses in seven patients (11.7%). However, the sources of infection remained unidentified in 18 patients (30.0%). Positive cultures were obtained from blood or vitreous samples in 38 patients (63.3%), yielding bacteria as the main organisms in 34 cases (56.7%), predominantly gram-negative organisms in 23 cases (67.6%), and gram-positive organisms in 11 (32.4%). Fungal infection was seen in four cases (6.7%). *Klebsiella *species were prevalent in our study, comprised 14 cases (22.7%), and were isolated in five out of seven cases of endogenous endophthalmitis, where the liver abscess was identified as the source of infection.

**Table 2:** Microorganism and source of infection crosstabulation (n = 60) The data have been represented as number (n) and percentage (%). STI, soft tissue infection

Microorganisms	Sources of infection, n (%)								
	Unknown	Genitourinary	Pneumonia	STI	Liver abscess	Endocarditis	Meningoencephalopathy	Septic arthritis	Parotid abscess
No growth	12 (20.0)	4 (6.7)	2 (3.3)	2 (3.3)	1 (1.7)	1 (1.7)	0 (0)	0 (0)	0 (0)
Klebsiella pneumoniae	1 (1.7)	3 (5.0)	3 (5.0)	0 (0)	5 (8.3)	0 (0)	0 (0)	0 (0)	1 (1.7)
Staphylococcus aureus	1 (1.7)	1 (1.7)	2 (3.3)	6 (10.0)	0 (0)	0 (0)	0 (0)	1 (1.7)	0 (0)
Escherichia coli	1 (1.7)	3 (5.0)	0 (0)	0 (0)	0 (0)	0 (0)	0 (0)	0 (0)	0 (0)
Candida albicans	0 (0)	2 (3.3)	0 (0)	0 (0)	0 (0)	0 (0)	1 (1.7)	0 (0)	0 (0)
Pseudomonas aeruginosa	0 (0)	1 (1.7)	1 (1.7)	0 (0)	1 (1.7)	0 (0)	0 (0)	0 (0)	0 (0)
Enterobacter cloacae	1 (1.7)	0 (0)	0 (0)	0 (0)	0 (0)	0 (0)	0 (0)	0 (0)	0 (0)
Acinetobacter lwoffii	1 (1.7)	0 (0)	0 (0)	0 (0)	0 (0)	0 (0)	0 (0)	0 (0)	0 (0)
Klebsiella ozanae	1 (1.7)	0 (0)	0 (0)	0 (0)	0 (0)	0 (0)	0 (0)	0 (0)	0 (0)
Aspergillus	1 (1.7)	0 (0)	0 (0)	0 (0)	0 (0)	0 (0)	0 (0)	0 (0)	0 (0)

Upon presentation, 56 eyes (82.4%) presented with visual acuity worse than 3/60. Three eyes (4.4%) perceived 6/60-3/60, four eyes (5.9%) perceived 6/18-6/36, and only five eyes (7.4%) presented with visual acuity of better than 6/12. Treatment involved vitreous tapping with intravitreal antibiotics and systemic antibiotic therapy upon diagnosis. All eyes received intravitreal antibiotics, and 36 eyes (52.9%) underwent vitrectomy. Eventually, 53 eyes (77.9%) had final visual acuity worse than 3/60, whereas only nine eyes (13.2%) achieved 6/12 or better post-treatment. Based on our results, there was a strong association between presenting visual acuity worse than 3/60 and blindness among patients with endogenous endophthalmitis (*p* = 0.010). However, other factors, such as age, gender, underlying comorbidities, microbial profiles, and vitrectomy, did not affect the final visual outcome (*p* > 0.05). The above data are presented in Table 3 and Table 4.

**Table 3:** Factors associated with blind visual outcome among the study population The data have been represented as number (n), percentage (%), crude odd ratio (crude OR), 95% confidence interval (95% CI), and p-value. *The *p* < 0.05 is considered statistically significant. DM, diabetes mellitus; ESRF, end-stage renal failure; HPT, hypertension; NA, not applicable

Variable	Visual outcome		Simple logistic regression		
	Blind [n (%)]	3/60 or better [n (%)]	Crude OR	95% CI	*p*-value
Age (n = 60)					
20-39	4 (66.7)	2 (33.3)	1	NA	NA
40-59	29 (74.4)	10 (25.6)	1.27	0.20, 7.97	0.797
60 and above	20 (87.0)	3 (13.0)	2.38	0.32, 17.74	2.375
Gender (n = 60)					
Male	25 (75.8)	8 (24.2)	1	NA	NA
Female	28 (80.0)	7 (20.0)	0.51	0.16, 1.64	0.260
DM (n = 60)					
No	5 (62.5)	3 (37.5)	1	NA	NA
Yes	46 (76.7)	14 (23.3)	1.97	0.42, 9.30	0.391
ESRF (n = 60)					
No	47 (74.6)	16 (25.4)	1	NA	NA
Yes	4 (80.0)	1 (20.0)	0.88	0.09, 8.47	0.908
HPT (n = 60)					
No	32 (76.2)	10 (23.8)	1	NA	NA
Yes	19 (73.1)	7 (26.9)	0.76	0.23, 2.55	0.660
Type of microbial (n = 68)					
No growth	19 (82.6)	4 (17.4)	1	NA	NA
Bacteria	32 (80.0)	8 (20.0)	1.41	0.42, 4.74	0.577
Fungus	2 (40.0)	3 (60.0)	0.24	0.03, 1.77	0.160
Presenting visual acuity (n = 68)					
6/6-6/12	1 (20.0)	4 (80.0)	1	NA	NA
6/18-6/36	1 (25.0)	3 (75.0)	1.33	0.06, 31.12	0.858
6/60-3/60	2 (66.7)	1 (33.3)	8	0.31, 206.37	0.210
2/60 and worse	49 (87.5)	7 (12.5)	20.89	2.09, 209.27	0.010*
Vitrectomy (n = 68)					
No	22 (68.8)	10 (31.3)	1	NA	NA
Yes	29 (80.6)	7 (19.4)	1.38	0.44, 4.36	0.58

**Table 4:** Presenting visual acuity association with blind visual outcome among the study population (n = 68) The data has been represented as number (n), percentage (%), adjusted odd ratio (Adj. OR), 95% confidence interval (95% CI), and p-value. *The *p* < 0.05 is considered statistically significant.

Variable	Visual outcome		Multiple logistic regression		
	Blind [n (%)]	3/60 or better [n (%)]	Adj. OR	95% CI	p-value
Presenting visual acuity					
2/60 and worse	49 (87.5)	7 (12.5)	20.89	2.09, 209.27	0.010*

Upon analyzing the final visual acuity in relation to the microbial profile, the lowest improvement in visual acuity was observed in eyes infected with gram-negative organisms following treatment (2, 2.9%) in contrast to eyes infected with gram-positive organisms (6, 8.8%) and fungi (3, 4.4%), or those with negative cultures (4, 5.9%). In fact, final visual acuity mostly deteriorates in those cases despite treatments (15, 22.0%). These data are summarized in Table 5.

**Table 5:** Final visual acuity status based on etiology (n = 68) The data have been represented as number (n) and percentage (%).

Organism	n (%)
Gram-positive organisms	
Improvement	6 (8.8)
No change	4 (5.9)
Deterioration	4 (5.9)
Gram-negative organisms	
Improvement	2 (2.9)
No change	6 (8.8)
Deterioration	15 (22.0)
Fungi	
Improvement	3 (4.4)
No change	1 (1.5)
Deterioration	1 (1.5)
Culture negative	
Improvement	4 (5.9)
No change	11 (16.2)
Deterioration	11 (16.2)

Five out of seven patients (71.4%) of endogenous endophthalmitis secondary to liver abscess had *Klebsiella pneumoniae* as the isolated organism. *Klebsiella pneumoniae* endogenous endophthalmitis was significantly correlated to the liver abscesses (*p* < 0.01), as depicted in Table 6. The correlation between visual acuity at presentation and final visual acuity is statistically significant (*r* = 0.707, *p* <0.01), as shown in Table 7. The blind visual outcome in this series was not shown to be statistically significantly related to patients’ age, sex, comorbidities, and microbial profile (i.e., gram-positive, gram-negative bacteria, and fungi).

**Table 6:** Association between Klebsiella pneumoniae endophthalmitis and liver abscess (n = 60) The data have been represented as number (n), percentage (%), odd ratio (OR), 95% confidence interval (95% CI), Pearson chi-square test (χ2 statistic), degrees of freedom (*df*), and p-value. *The *p* < 0.05 is considered statistically significant. NA, not applicable

*Klebsiella pneumoniae* isolates	Liver abscess [n (%)]		OR (95% CI)	χ^2 ^statistic (*df*)	p-value
	Yes	No			
Yes	5 (8.3)	13 (21.7)	27.27 (2.98, 249.94)	15.34 (1)	<0.001*
No	2 (3.3)	40 (66.7)	NA		

**Table 7:** Correlation between presenting visual acuity and final visual acuity in logMAR (n = 68) The data have been represented as Pearson’s correlation coefficient (*r*) and *p*-value. *The *p* < 0.05 is considered statistically significant.

Presenting visual acuity	Final visual acuity	
	Pearson’s correlation (*r*)	*p*-value
	0.707	<0.001*

## Discussion

There have been limited reviews of endogenous endophthalmitis in Asia, particularly in Southeast Asian countries [1,6,8,9]. In 2018, Muda et al. and Michael et al. described reports on endogenous endophthalmitis in Malaysia, while Silpa-Archa reported data from Thailand, but all their data were only from a single tertiary center [1,8]. Wong et al. conducted a study involving three major public hospitals in Singapore. However, this was old data back in 2000 [6]. Table 8 summarizes published reports on endogenous endophthalmitis from Asian countries (India, Thailand, Singapore, and Malaysia), including two neighboring Oceanian countries (New Zealand and Australia) [2,6,8,9,12-14].

**Table 8:** Comparison of published studies on endogenous endophthalmitis The data have been represented as number (n), percentage (%), and mean ± SD. MRSA, methicillin-resistant *Staphylococcus aureus*; NA, not applicable

Variable	n (%)/mean ± SD							
	Present study	Michael et al. [9]	Silpa-Archa et al. [8]	Wong et al. [6]	Nishida et al. [2]	Ratra et al. [12]	Samalia PD et al. [13]	Gounder et al. [14]
Country; year	Malaysia; 2024	Malaysia; 2018	Thailand; 2018	Singapore; 2000	Japan; 2015	India; 2015	New Zealand; 2020	Australia; 2020
No. of eye center	2	1	1	3	3	1	1	3
Population	60	17	36	27	21	58	62	57
No. of eyes	68	18	41	32	27	61	78	66
Mean age in years	54.9±13.3	53.2±13.2	58	50	68.5±9.7	34.6±14.9	61.6	53
Gender								
Male	27 (45.0)	7 (41.2)	23 (64.0)	19 (70.4)	14 (66.7)	36 (62.1)	32 (51.6)	(61.4)
Female	33 (55.0)	10 (58.8)	13 (36.0)	8 (29.6)	7 (33.3)	22 (37.9)	30 (48.4)	(38.6)
Laterality								
Right	25 (41.7)	4 (23.5)	15 (35.6)	14 (51.9)	5 (23.8)	35 (57.4)	33 (42.3)	17 (25.8)
Left	27 (45.0)	12 (70.6)	16 (39.20	7 (25.9)	10 (47.6)	26 (42.6)	31 (39.7)	31 (47.0)
Both	8 (13.3)	2 (5.9)	5 (12.2)	6 (22.2)	6 (28.6)	3 (5.2)	14 (22.6)	18 (27.2)
Risk factor								
DM	53 (88.3)	15 (88.2)	11 (30.6)	11 (40.7)	13 (61.9)	14 (24.1)	24 (38.7)	19 (33.0)
HPT	24 (40.0)	12 (70.6)	8 (22.2)	NA	6 (28.6	NA	NA	NA
IHD	24 (40.0)	3 (17.6)	NA	NA	5 (28.8)	NA	NA	NA
ESRF	5 (8.3)	3 (17.6)	1 (2.8)	NA	NA	2 (3.4)	16 (25.8)	NA
Malignancy	NA	NA	NA	NA	5 (28.8)	NA	17 (27.4)	6 (11.0)
Autoimmune disease	NA	NA	1 (2.8)	1 (3.7)	NA	NA	4 (6.5)	NA
IVDU	NA	NA	NA	NA	NA	1 (1.7)	NA	17 (30.0)
Presenting visual acuity								
6/6-6/12	5 (7.4)	0 (0)	1 (2.7)	4 (12.5)	5 (20.0)	0 (0)	NA	NA
6/18-6/36	4 (5.9)	0 (0)	1 (2.7)	5 (15.6)	5 (20.0)	5 (8.2)	NA	NA
6/60-3/60	3 (4.4)	1 (5.6)	3 (8.3)	10 (31.3)	4 (16.0)	10 (16.4)	NA	NA
2/60 and worse	56 (82.4)	17 (94.4)	31 (86.1)	13 (40.6)	11 (44.0)	46 (75.4)	NA	NA
Final visual acuity								
6/6-6/12	9 (13.2)	1 (5.6)	4 (11.1)	8 (25.0)	11 (44.0)	7 (11.5)	NA	NA
6/18-6/36	5 (7.4)	3 (16.7)	1 (2.7)	1 (3.1)	5 (20.0)	4 (6.6)	NA	NA
6/60-3/60	1 (1.5)	0	2 (5.6)	0 (0.0)	4 (16.0)	7 (11.5)	NA	NA
2/60 and worse	53 (77.9)	14 (77.8)	29 (80.6)	23 (71.9)	5 (20.0)	33 (54.1)	NA	NA
Treatment								
Medical only	32 (47.1)	8 (47.1)	12 (33.3)	22 (68.7)	21 (77.8)	11 (18.0)	18 (29.0)	37 (56.0)
Surgical intervention	36 (52.9)	9 (52.9)	24 (66.7)	10 (31.3)	6 (22.2)	38 (62.3)	44 (71.0)	29 (44.0)
Microorganism								
No growth	22 (36.7)	7 (41.2)	0 (0)	7 (21.9)	2 (9.5)	27 (44.3)	0 (0)	4 (6.1)
Bacteria	34 (56.7)	10 (58.8)	35 (97.2)	25 (69.4)	21 (100)	29 (85.3)	57 (73.1)	30 (57.0)
Gram-positive	11 (32.4)	3 (17.6)	14 (38.9)	6 (18.8)	16 (76.2)	9 (26.5)	40 (51.3)	19 (35.8)
*Staphylococcus aureus*	11 (18.3)	2 (11.8)	2 (5.6)	4 (12.5)	12 (57.1)	3 (5.2)	19 (24.4)	10 (15.2)
MRSA	NA	NA	NA	NA	8 (38.1)	NA	5 (6.4)	NA
*Streptococcus *sp.	NA	NA	12 (33.3)	2 (6.3)	5 (23.8)	1 (1.7)	14 (23.1)	10 (15.2)
Gram-negative	23 (67.6)	7 (41.2)	16 (44.4)	19 (59.4)	4 (19.0)	20 (58.8)	17 (21.8)	11 (20.8)
Klebsiella pneumoniae	13 (22.7)	3 (17.6)	9 (25.0)	18 (56.3)	2 (9.5)	1 (1.7)	8 (10.3)	9 (13.6)
Escherichia coli	4 (6.7)	NA	4 (11.1)	1 (3.1)	NA	4 (6.9)	6 (7.7)	1 (1.5)
Pseudomonas aeruginosa	3 (4.4)	3 (17.6)	2 (5.6)	NA	NA	8 (13.8)	NA	1 (1.5)
*Enterobacter *sp.	1 (1.7)	1 (1.7)	NA	NA	1 (4.8)	NA	NA	NA
*Acinobacter *sp.	1 (1.7)	NA	NA	NA	NA	NA	NA	NA
*Klebsiella ozanae*	1 (1.7)	NA	1 (2.8)	NA	NA	5 (14.7)	NA	NA
Fungi	4 (6.7)	NA	1 (2.8)	NA	NA	3 (5.2)	21 (26.9)	19 (35.8)
*Candida *sp.	3 (4.4)	NA	NA	NA	NA	3 (5.2)	8 (10.3)	15 (22.7)
*Aspergillus*	1 (1.7)	NA	NA	NA	NA	NA	7 (9.0)	1 (1.5)
Source of infection								
Unknown	19 (31.7)	5 (29.4)	13 (36.1)	2 (7.4)	4 (19.0)	27 (46.5)	9 (14.5)	0 (0)
Genitourinary	14 (22.7)	5 (29.4)	5 (13.9)	1 (3.7)	1 (4.8)	3 (8.6)	11 (17.7)	16 (28.0)
Pneumonia	8 (13.3)	3 (17.6)	5 (13.9)	3 (11.1)	2 (9.5)	2 (3.4)	11 (17.7)	13 (23.0)
Soft tissue infection	8 (13.3)	1 (5.9)	2 (5.6)	1 (3.7)	3 (14.3)	2 (3.4)	NA	5 (9.0)
Hepatobiliary infection	7 (10.5)	2 (11.8)	5 (13.9)	16 (59.3)	1 (4.8)	1 (1.7)	NA	23 (41.0)
Infective endocarditis	1 (1.7)	NA	NA	NA	3 (14.3)	NA	10 (16.1)	12 (21.0)
Meningoencephalitis	1 (1.7)	NA	3 (8.3)	1 (3.7)	NA	1 (1.7)	NA	4 (7.0)
Bone and joint infection	1 (1.7)	NA	3 (8.3)	1 (3.7)	NA	NA	NA	7 (12.0)
Parotid abscess	1 (1.7)	NA	NA	NA	NA	NA	NA	NA
Gastrointestinal	NA	NA	1 (2.8)	2 (7.4)	2 (9.5)	10 (17.2)	5 (8.1)	1 (2.0)
Catheter-related infection	NA	1 (5.9)	NA	NA	2 (9.5)	NA	11 (17.7)	NA

In our study, the mean age at presentation was 54.9 ± 13.3 years, consistent with findings indicating a peak incidence during the fifth decade of life [1,3-6,8,9,14-20]. Despite certain previous reports of a male predominance [2-4,6,8,14,16,19,21,22], our study observed a higher incidence among women (55.0%), which tallied with studies reported by Michael et al. and Namvar et al. [9,23]. Left-eye involvement was observed in 58.3% of our patients. Previous research suggested a higher occurrence of endogenous endophthalmitis in the right eye due to direct arterial blood flow from the right carotid artery [7]. However, recent reviews have indicated a higher prevalence in the left eye, indicating that carotid vessel anatomy may have a minimal impact on endogenous endophthalmitis location [2-4].

Diabetes mellitus was the most prevalent predisposing factor, affecting 88.3% of our patients. This finding was consistent with most reported studies [1,3-6,9,13-15,18-22,24]. In contrast, Callegan et al. and Connell et al. reported that intravenous drug abuse was the most common predisposing factor instead of diabetes mellitus [20,25]. Most of our patients had identifiable sources of infection, with genitourinary infections and liver abscesses being the most common at 23.3% and 11.7%, respectively, similar to other published data [1,5,8,9,14,18,21]. Other studies also reported a high prevalence of catheter-related infections, with infective endocarditis being the most prominent source of infection [13,14].

Bacteria were predominant in our study (34, 56.7%), and gram-negative organisms were more prevalent (23, 67.6%). This observation was in keeping with studies that were mainly conducted in Asia [1,6,8,9,12,19]. In contrast, studies conducted in Western countries showed predominant fungal isolates [3,5,17,20,24,25]. We hypothesize that the difference in pathogens is probably attributable to geographical and climate distinctions. *Klebsiella *infection has been recognized as a prevalent cause of endogenous endophthalmitis throughout Asia [1,6,8,9,19,22]. Similar to our study, *Klebsiella pneumoniae* infection stood out as the most common, representing 22.7% of cases. Nonetheless, instances of *Klebsiella *endogenous endophthalmitis have surfaced in Western nations since the mid-1990s, with the incidence steadily rising in recent years [4,26-28].

Our study demonstrated a poor visual outcome in endogenous endophthalmitis cases, particularly in cases of gram-negative infections, where 22% of eyes showed visual deterioration post-treatment. This aligns with other data indicating that gram-negative infections tend to lead to a poorer visual prognosis compared to gram-positive infections [4,9,14,16,21,22]. This could be due to the rapid progression of disease contributed by its virulence factors, higher level of antibiotic resistance, and robust inflammatory response. In contrast, other reported literature, particularly from Western countries, found that fungal isolates significantly lead to a poor visual outcome as compared to bacterial endogenous endophthalmitis [3,15-17,24,25].

In 71.4% of cases of endogenous endophthalmitis secondary to liver abscess, we observed that *Klebsiella pneumoniae* were found to be positive, consistent with other published studies [19,21,22,27], suggesting close monitoring of patients with such infections. With regards to the visual outcome, we did not find any significant association between *Klebsiella* species and poor visual outcomes, in contrast with Chen et al. and Ghiam et al. [21,28]. Liver abscesses are associated with *Klebsiella pneumoniae* due to its ability to trigger a metastatic infection, particularly in individuals with conditions such as diabetes or chronic liver disease. Additionally, some *Klebsiella strains* have a protective capsule that helps them avoid the host's immune responses. This will let the organism stay in liver tissues and make abscesses grow even faster [29]. Patients with *Klebsiella *endogenous endophthalmitis, therefore, should be evaluated for a concurrent liver abscess, as the two conditions have been proven to be associated. Early detection and treatment of the liver abscess can help prevent complications related to the spread of infection, thereby avoiding the occurrence of endogenous endophthalmitis.

Limited information exists regarding prognostic markers, primarily derived from small case series. In this study, only poor initial visual acuity emerged as a significant risk factor for a blind visual outcome. These findings align with prior research, particularly in Asian populations, where poor initial visual acuity was similarly linked to unfavorable outcomes in smaller studies [8,19]. For instance, a larger study in Taiwan involving 86 subjects revealed a significant correlation between poorer vision than counting fingers at presentation and poor visual outcomes [21]. Similarly, a recent investigation in Western Australia indicated that baseline visual acuity served as a predictor for final visual acuity [14].

Although infections caused by gram-negative organisms in our series were associated with the most deterioration in vision, simple univariate analysis did not show that such microbial profiles were significantly related to blind visual outcomes. This lack of statistical significance in the association between visual outcome and the type of infecting organism in our series may be attributed to the small number of patients involved. A larger sample size and a longer duration of study are needed to investigate the relationship further. Additionally, different statistical methods could be employed to potentially reveal a significant association.

We observed that doing a vitrectomy did not, however, correlate with favorable visual outcomes (p = 0.58), in contrast with Conneli et al., who found that vitrectomy did improve visual outcomes, especially when it was done for bacterial-proven cases [20]. Our results support those of Cho et al., who looked at 128 eyes of 108 patients with endogenous endophthalmitis at two referral centers in Boston, USA, and Seoul, South Korea, from 2006 to 2013. They found that vitrectomy did not have a statistically significant effect on the final vision of the whole group [30]. We postulate that this can be due to the late patient’s presentation with poor presenting visual acuity, which leads to severe retinal insult, resulting in poor vision post-vitrectomy. This study is limited by a small sample size from only the East Coast of Malaysia, which may not be representative of the entire Malaysian population. It is also constrained by the absence of clinical data regarding the antimicrobials used in each case and uncertainty about the timing of the vitrectomy, which could potentially impact the visual outcomes post-surgery. Moving forward, we need additional detailed data on the mentioned points to verify and expand our conclusions.

## Conclusions

Endogenous endophthalmitis poses a significant risk, not only to eyesight but also to potentially life-threatening systemic consequences. Females were found to be more commonly affected by endogenous endophthalmitis than males. The visual prognosis is often unfavorable, especially in situations involving gram-negative bacterial infection. *Klebsiella *species were the most common isolated microorganisms, and *Klebsiella pneumoniae* was commonly associated with liver abscesses, which often resulted in poor visual outcomes. Urinary tract infection was the most common predisposing factor. A majority of the patients had poor presenting and final visual acuity, which is a significant indicator of blind visual outcomes. Hence, a high index of suspicion, early diagnosis, and treatment are crucial to salvaging useful vision.
